# Effect of Heat-Moisture Treatment on the Physicochemical Properties and Starch Digestibility of Mix Powder (Wheat Flour-Black Soybean Flour) and Corresponding Cookies

**DOI:** 10.3390/gels8070429

**Published:** 2022-07-09

**Authors:** Liping Yang, Sunyan Wang, Songnan Li, Gongqi Zhao, Chuanlai Du

**Affiliations:** 1 School of Food Engineering, Anhui Science and Technology University, 9 Donghua Road, Chuzhou 233100, China; sy18856427414@163.com (S.W.); zhaogongqi2022@163.com (G.Z.); 2 Joint International Research Laboratory of Agriculture and Agri-Product Safety, Institutes of Agricultural Science and Technology, Yangzhou University, 48 Wenhui East Road, Yangzhou 225009, China; lsnyz2020@yzu.edu.cn

**Keywords:** heat-moisture treatment, superfine black soybean flour, starch digestibility, cookies

## Abstract

In order to improve the nutritional value and reduce starch the digestibility of black soybean cookies, superfine black soybean flour was modified by heat-moisture treatment (HMT). The physicochemical properties, structure analysis of the flour samples and corresponding dough, and nutritional, physical, and textural properties of the cookies were investigated. After HMT, the water and lactic acid retention capacity and the oil binding capacity of mix powder dramatically increased, being almost twice the value of the untreated sample. HMT increased gelatinization temperature by about 10 °C but decreased gelatinization enthalpy. HMT had no apparent effect on the morphology and size of granules, but some cracks and pores appeared on the HMT-mix powder granules and corresponding dough. Fourier transform infrared spectroscopy analysis showed that the ordered structure of dough was unaffected during HMT. After HMT, the thickness, density, and baking loss of the cookies increased, and the spread ratio decreased. HMT dramatically increased the chemical score of cookies from 12.35% in mix powder cookies to 19.64% in HMT-mix powder cookies. HMT decreased the rapidly digestible starch content, while the slowly digestible starch increased from 45.97% in mix powder cookies to 49.31% in HMT-mix powder cookies, and RS increased from 21.64% to 26.87%. Overall, HMT did not have a negative effect on the processing properties and microstructure and secondary structure of the dough, or the physical properties and quality of the cookies, but significantly improved the nutritional properties and decreased the starch digestibility of the cookies.

## 1. Introduction

Cookies are a popular bakery product for most consumers, including children and the elderly. Its popularity is due to its ready-to-eat nature, convenience, rich shapes and tastes, relatively longer shelf-life, and ability to serve as a vehicle for various nutrients and supplements [1,2,3]. However, cookies usually contain a high amount of fat and sugar, leading to high calories. Moreover, most cookies are made from refined wheat flour with a low nutrient density. The most typical nutrient deficiencies are lysine and dietary fiber [4]. With the increasing awareness of the link between diet and health, health and conveniences are the two major factors in the current development of snacks and various products [5]. Thus, multigrain foods rich in dietary fiber have become a new trend in improving the nutritional value of food [6,7].

Black soybean is an important variety of coarse grains that contains rich dietary fibers, proteins, vitamins, micronutrients, and lysine. It has many physiological functions, including antioxidative capacity, risk reduction in obesity, diabetes, hypercholesterolemia, coronary heart diseases, and other diseases [5,8]. Thus, the application of black soybean in cookies is of great significance in improving the nutrition of cookies. In previous studies, the effect of black soybean flour particle size on dough characteristics of mix powder and wheat flour and the digestibility, physicochemical and sensory characteristics of cookies were investigated. The results showed that the cookie samples containing coarse black soybean flour had lower rapidly digestible starch (RDS) and higher slowly digestible starch (SDS) and resistant starch (RS). Based on the physicochemical quality and sensory evaluation of the cookies, the superfine black soybean flour was more suitable for producing high-quality cookies. Thus, how to enhance the RDS and RS content of superfine black soybean flour cookies needs to be further studied, thus achieving higher sensory quality and nutritional characteristics of black soybean cookies.

Heat-moisture treatment (HMT) is a physical modification method used to change the physicochemical properties of starch. It is the process in which the starch is heat-treated at a low moisture content and a relatively high temperature [9]. Many studies have reported that the structural and physicochemical properties of starches are affected by HMT. In addition, HMT directly affects the digestibility of starches through SDS and RS formation and reduction in RDS, which is extremely important for providing health benefits to consumers. Rizkalla [10] found that consumption of foods rich in SDS and RS resulted in a low GI diet, which was beneficial to improving glycemic control and decreasing the incidence and prevalence of diabetes and cardiovascular disease. These benefits could be attributed to the effects of SDS and RS on satiety, physical performance, lower blood lipid levels, and insulin resistance, as they reduce the stress on regulatory systems related to glucose homeostasis [11]. To meet the health requirements of consumers, it is necessary to develop foods rich in SDS and RS contents. Many studies have examined the variations in starch digestibility and the physicochemical properties before and after HMT. Na et al. [12] revealed that the hydrothermal treatment of sweet potato starch significantly increased the SDS and RS content. Tan et al. [9] reported that the SDS content of HMT-breadfruit starch was 13.24%, which was 10.25% higher than that of native starch. Furthermore, their study proposed that the increased enzyme resistance may be attributed to a more compact granule structure and the rearrangement of molecular chains. However, there are limited studies on the increasing SDS and RS of mix powder (wheat flour-black soybean flour) by HMT. Detailed reports on the incorporation of HMT-mix powder in cookies are also lacking, and how the HMT affected the processing characteristics of the mix powder and the physicochemical properties of cookies is still unknown, limiting the application of HMT on mix powder of wheat flour and black soybean flour.

Thus, in this study, HMT was used to process mix powder of wheat flour and superfine black soybean powder. The physicochemical properties of mix powder and the structure of the dough were analyzed, as well as the physical characteristics, texture, in vitro starch digestibility, and nutritional properties of the cookies. This study aims to decrease the starch digestibility of black soybean cookies, while maintaining the texture of the cookies, which is of great significance to consumers of obesity diseases and other chronic diseases. The results can provide theoretical guidance for the deep processing of black soybeans and the development of high-quality and low-digestibility black soybean cookies with HMT-black soybean flour.

## 2. Materials and Methods

### 2.1. Materials

Wheat flour and black soybeans were purchased from a local market, and the superfine black soybean flour was prepared using a high-energy nano-impact mill (CJM-SY-B, Qinhuangdao Taiji Ring Nano-Products Co., Ltd., Qinhuangdao, China). Mix powder was obtained by replacing wheat flour with 35% superfine black soybean flour. The porcine pancreas α-amylase (P7545, 8 × USP) and amyloglucosidase (A7095 ≥ 300 U/mL) were obtained from Sigma–Aldrich Co. (St. Louis, MO, USA). All other chemicals were of analytical grade.

### 2.2. Heat-Moisture Treatment (HMT)

The HMT of wheat and black soybean mix powder was conducted referring to Wang et al. [13] with some modifications. The moisture level of the mix powder sample was adjusted to 25%, and the sample was equilibrated at 4 °C for 24 h. Then, 30 g of the sample was sealed in a 500 mL screwed stainless-steel reactor. The reactor was transferred into an oil bath equipped with a magnetic stirrer, and the HMT was conducted at 120 °C for 4 h, with continuous stirring to ensure uniform heating. After cooling, the HMT-modified mix powder sample was removed from the containers and subsequently dried at 40 °C to achieve a uniform moisture content. The HMT-modified mix powder sample was obtained after grinding and was labeled as HMT-mix powder.

### 2.3. Proximate Composition Analysis

The ash, total protein, fat, and total dietary fiber content of wheat flour, mix powder, and HMT-mix powder were determined using standard AACC (2000) methods [14]. The nitrogen content was determined using an automatic Kieldahl apparatus (Kjeltec™ 8400, Foss Inc., Hoganas, Sweden), and the nitrogen conversion coefficient was set to 6.25. A moisture analyzer (MA35, Sartorius Stedim Biotech GmbH, Goettingen, Germany) was used to analyze the moisture content of the samples.

### 2.4. X-ray Diffraction and Relative Crystallinity

X-ray diffraction patterns of flour samples were obtained with the X-ray diffractometer (TTRIII, Rigaku, Japan) at 40 kV and 80 mA. The diffraction angle (2θ) scanning region range was 4°–50°, and the rate was 1°/min. The crystalline peak area and amorphous area were separated by PeakFit software (Version 4.12, Systat Software Inc., San Jose, CA, USA). The relative crystallinity of the samples was calculated as the ratio of the crystalline peak area to the total diffraction area [15].

### 2.5. Water Retention Capacity, Lactic Acid Retention Capacity, and Oil Binding Capacity

The water retention capacity (WRC), lactic acid retention capacity (LARC), and oil binding capacity (OBC) of wheat flour, mix powder, and HMT-mix powder were determined by the method described by Cappa et al. [8]. Briefly, 3 g of mixed powder samples were weighed and transferred to a 50 mL centrifuge tube. Next, 30 mL of water, lactic acid (5.0%, *w*/*w*), and maize oil were added to determine the WRC, LARC, and OBC, respectively. The mixtures were vigorously shaken for 10 s. After that, the tubes were incubated in a 30 °C water bath for 30 min, shaken for 5 s every 10 min, and then centrifuged at 3000× *g* for 10 min to remove the supernatant. Centrifuge tubes with precipitate were weighed, and the WRC, LARC, and OBC values were calculated as the weight of the solvent contained in the sample. The formula is as follows:

(1) $$\mathrm{WRC}/\mathrm{LARC}/\mathrm{OBC}\;\left(g/g\right)=\frac{Precipitate\;weight\left(g\right)-Sample\;weight\left(g\right)}{Sample\;weight\left(g\right)}$$

### 2.6. Thermal Properties

The thermal properties of the wheat flour, mix powder, and HMT-mix powder were measured using a differential scanning calorimeter (Model DSC3, Mettler-Toledo, Greifensee, Switzerland). The method was referred to Yang et al. [15] with some modifications. Briefly, 3 mg of starch and 9 μL of distilled water were added into an aluminum pan and hermetically sealed. After equilibration at room temperature for 24 h, the samples were heated from 20 °C to 120 °C at 5 °C min^−1^ using an empty pan. The gelatinization temperature at onset (To), peak (Tp), and end (Tc) and gelatinization enthalpy (ΔH) were calculated based on the thermogram.

### 2.7. Scanning Electron Microscopy (SEM)

The surface morphology of wheat flour, mix powder, HMT-mix powder, and their doughs were observed under cold field-emission scanning electron microscopy (EVO-18, Carl Zeiss, Jena, Germany). For the dough preparation, wheat flour, mix powder, and HMT-mix powder were separately mixed with water, and the dough was prepared by stirring the flour at 50 rpm for 10 min in a dough mixer (SJJ-D08G1, Little Bear Electric Appliance Co., Ltd., Guangdong, China). Then, the dough was freeze-dried and cut into small pieces [16]. The flour and dough samples were attached to double-sided tape mounted on an aluminum stub and sputter-coated with a thin layer of platinum under a vacuum. The examination was conducted at an accelerating voltage of 20.0 kV.

### 2.8. Determination of Free Sulfhydryl Groups and Disulfide Bonds

The determination of free sulfhydryl groups (-SH) in the gluten was performed according to the spectrophotometric assay method reported by Bressiani et al. [17] and Cao et al. [18]. The dough sample was freeze-dried and ground (75 mg) to determine the content of free SH and total SH. The contents of free SH, total SH, and S–S were calculated using the following formulas:

(2) $$\mathrm{Free}\;\mathrm{SH}\left(\mu \mathrm{mol}/g\right)=73.53A_{412}\times D_{1}/C\;$$

(3) $$\mathrm{Total}\;\mathrm{SH}\left(\mu \mathrm{mol}/g\right)=73.53A_{412}\times D_{2}/C\;$$

(4) $$S-S\left(\frac{\mu \mathrm{mol}}{g}\right)=\frac{\mathrm{total}\;\mathrm{SH}-\mathrm{free}\;\mathrm{SH}}{2}\;$$

where *A*_412_ means the absorbance at 412 nm; *D* means the dilution factor (*D*_1_ is 5.02 and *D*_2_ is 10); *C* denotes the sample concentration in mg/mL.

### 2.9. Fourier Transform Infrared Spectroscopy (FTIR) Analysis

The secondary structures of the gluten were studied by an FTIR spectrometer (IS50, Thermo Nicolet Corporation, Waltham, MA, USA), according to the method described by Xu et al. [16]. After lyophilization and grinding, the powdered samples were mixed with anhydrous KBr (1:100, *w*/*w*) and compressed into thin disk-shaped pellets. FTIR spectra were recorded from 4000 to 400 cm^−1^ with a resolution of 4 cm^−1^.

### 2.10. Preparation of Cookies

Cookies were separately prepared using wheat flour, mix powder, and HMT-mix powder. The cookies were produced using the method reported by Sulieman et al. [19] with a slight modification. The basic dough formula (based on 100 g of flour) for making cookies was as follows: 16.0 mL of water, 20.0 g of sugar, 14.0 g of vegetable oil, 14.0 g of margarine, 0.3 g of salt, 0.3 g of ammonium bicarbonate, and 0.2 g of sodium bicarbonate. All baking ingredients and water were weighed into a stainless-steel bowl and manually mixed for 15 min to obtain a homogeneous cream system. Then, all the flour was subsequently added and mixed in a 300 g pin mixer (Brabender, Duisburg, Germany) at 25 °C for 5 min at the speed of 6000 rpm to form a homogenous dough. The dough was pressed into 3.0 mm thickness pieces and rotated and shaped in circular molds with 65 mm diameter and 3.5 mm depth. Cookies were baked in an oven (Mondial Forni, Verona, Italy) at 200 °C for 13 min, then cooled to room temperature and packaged in sealed polyethylene bags for further physicochemical, structure, and sensory analysis.

### 2.11. Amino Acid Composition and Evaluation

The amino acid composition of the cookie samples was measured by Amino Acids Automatic Analyzer (Hitachi Ltd. L-8900, Tokyo, Japan) following the method described by Zhao, Mu, and Sun [20] with few modifications. Briefly, 80 mg powdered cookie was mixed with 10 mL of 6 N HCl in a Pyrex test tube and subjected to nitrogen sweeping for 1 min. Then, the test tube was sealed and heated for 24 h at 110 °C to prepare the hydrolysate. The hydrolysate was filtered through 0.2 μm filter membranes and dried at 60 °C under vacuum conditions. The dry hydrolysate was dissolved in 1 mL of 0.02 N HCl and then analyzed with 20 μL of the solution. Protein quality was evaluated by amino acid score (AAS), which was calculated by comparing the essential amino acid (EAA) content in the cookies’ protein with the FAO/ WHO 2007 recommendations. The suggested levels of each EAA (mg/g) were as follows: lysine, 4.5; leucine, 5.9; valine, 3.9; histidine, 1.5; threonine, 2.3; isoleucine, 3.0; methionine, 1.6; phenylalanine, 3.0.

### 2.12. Physical Characteristics Evaluation of Cookies

The physical parameters of the cookies, such as weight, diameter, thickness, spread ratio, density, and baking loss, were determined according to the method reported by Sulieman et al. [19] and Hera et al. [21].

### 2.13. In Vitro Starch Digestibility

The cookies were ground into powders to determine the in vitro starch digestibility. The enzymatic hydrolysis of the cookies was determined as described by Yang et al. [15].

The fractions of RDS, SDS, and RS were calculated by the following formulas:

(5) RDS(%)=(G20−G0)×0.9×100

(6) SDS(%)=(G120−G20)×0.9×100

(7) RS(%)=100−RDS−SDS

where *G*20 represents the glucose released after 20 min; *G*120 represents glucose released after 120 min; *G*0 represents free glucose.

### 2.14. Textural Properties Measurements

The textural properties of the cookies were determined by texture profile analysis using a CT3 texture analyzer (Stable Micro systems Ltd., Godalming, UK) with a TA39 probe. The test parameters were set as follows: the pre-test speed, test speed, and post-test speed were 1 mm/s; the degree of compression was 50%; the dwell time between two compressions was 2 s, and cycled twice. The TextureLoader software was used for data collection and processing. The hardness, fracturability, gumminess, and chewiness were recorded.

### 2.15. Statistical Analysis

All analyses were conducted in triplicate using SPSS 17.0 (SPSS Inc., Chicago, IL, USA). Significant differences were determined by comparing the means using Duncan’s multiple range test at a significance level of *p* < 0.05.

## 3. Results and Discussion

### 3.1. Proximate Composition Analysis

The proximate composition of wheat flour, mix powder, and HMT-mix powder is presented in Table 1. The content of mix powder was significantly higher than in wheat flour. The content of moisture, ash, protein, lipid, and fiber was higher in mix powders than in wheat flour, especially the content of protein, lipid, and dietary fiber. The protein, lipid, and dietary fiber in mix powder were 2.64, 1.65, and 0.54 g·kg^−1^, respectively, while in wheat flour, they were 1.67, 0.90, and 0.13 g·kg^−1^, respectively. This result was mainly because of the characteristics of the raw materials. After HMT, some changes in the proximate composition of the HMT-mix powder occurred. In summary, the ash, protein, lipid, and dietary fiber all decreased after HMT. The decrease in these components may be due to degradation during the higher temperature treatment.

### 3.2. X-ray Diffraction and Relative Crystallinity

**Figure 1:**
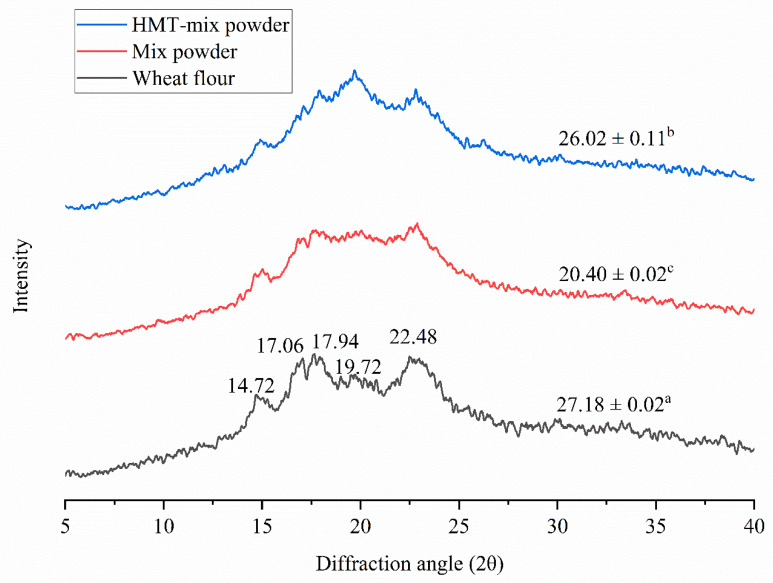
X-ray diffraction patterns of wheat flour, mix powder and HMT-mix powder. Numbers showed in mean ± SD values indicate the relative crystallinity. ^a–c^ Means in the same column with different lowercase letters were significantly different.

XRD assay was conducted to analyze the changes in the crystalline type and relative crystallinity of the mix powder after HMT. The X-ray diffractograms and relative crystallinities of wheat flour, mix powder, and HMT-mix powder are displayed in Figure 1. All the samples showed the typical A-type pattern with diffraction intensities at 15.0°, 17.0°, 17.9°, 19.72° and 22.8° 2θ angles, suggesting that the HMT did not change the crystal pattern. Compared to wheat flour, most of the diffraction peak patterns of mix powder and HMT-mix powder were attenuated to a certain extent, which may be caused by the dilution of starch with the mix of black soybean flour. However, the peak intensity at 20° dramatically increased. The result was consistent with the finding of Wang et al. [13], who observed that HMT-rice starch did not present any changes in the crystal type, except a slight reduction in the diffraction peak intensities, but the peak intensity at 20° presented an apparent increase. In addition, they pointed out that the significant increase in peak intensity 20° was mainly attributed to the formation of amylose–lipid complexes during HMT.

Although HMT did not change the crystal pattern, it increased the relative crystallinity. Moreover, the relative crystallinity significantly increased from 20.40% in mix powder to 26.02% in HMT-mix powder. This result is consistent with Na et al. [12] who found that the relative crystallinity of purple sweet potato increased from 26.6% to 29.7% after HMT. It has been suggested that several double helices are disrupted in the amorphous regions by HMT and that interactions between amylose chains also occur during the treatment [12]. The amorphous areas of starch are usually destroyed by HMT, which may increase relative crystallinity.

### 3.3. Water Retention Capacity, Lactic Acid Retention Capacity, and Oil Binding Capacity

The WRC, LARC, and OBC of powders need to be noticed when developing new products. In this study, these parameters of the wheat flour, mix powder, and HMT-mix powder are shown in Table 2. Compared to the wheat flour sample, the OBC and LARC of mix powder decreased due to the higher lipid content in black soybean, inhibiting the absorption and retention of oil and lactic acid. Nevertheless, the WRC of mix powder increased, which may be attributed to the higher fiber and protein content in black soybean, as WRC was significantly and positively correlated with protein content [8]. The WRC, LARC, and OBC of mix powder dramatically increased after HMT. The WRC LARC, and OBC of HMT-mix powder were 1.38, 1.70, and 1.50, respectively, almost twice as much as the value of mix powder. As the proximate components of the HMT-mix powder, such as protein and dietary fiber, did not show significant changes (as shown in Table 1), the change in WRC, LARC, and OBC after HMT may be related to the changes in other factors. The changes in particle and crystalline structure should be the main factors. Furthermore, HMT resulted in cracks on the powder particles (as shown in the following SEM analysis), facilitating the combination of the HMT-mix powder with water, oil, and lactic acid and showing a significant increase in WRC, LARC, and OBC.

### 3.4. Thermal Properties

**Figure 2:**
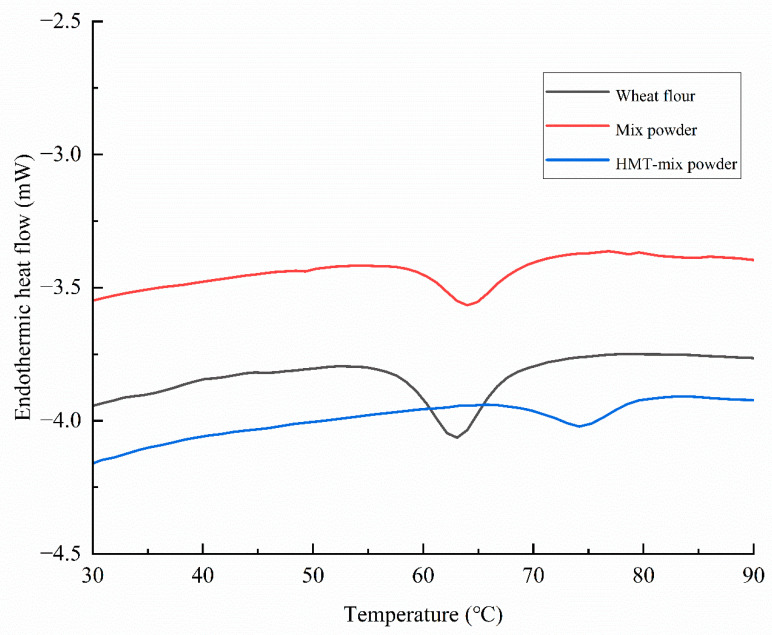
DSC curve of wheat flour, mix powder and HMT-mix powder.

DSC allows the analysis of transition temperatures and the transition enthalpies, corresponding to the melting of starch crystallites during heating. The DSC curves are showed in Figure 2. The thermal parameter onset (To), peak (Tp), and conclusion (Tc) temperatures, melting temperature range (Tc-To), and melting enthalpy (ΔH) of the samples are summarized in Table 3. Wheat flour and mix powder showed an endothermic peak at about 63 °C. In addition, the To, Tp, and Tc of mix powder were higher than that of wheat flour, which may be attributed to the higher protein and fiber of mix powder. The presence of protein and dietary fiber in the mix powder significantly enhanced the competition for water distribution between starch, protein, and dietary fiber components [22]. As a result, the gelatinization temperature of mixed powder increased. However, the gelatinization enthalpy values of mix powder significantly decreased with the addition of black soybean flour, from 5.28 J/g in wheat flour to 3.17 J/g in mix powder. This result agreed with the findings of Sozer et al. [4] who found that the enthalpy values decreased by half when flour samples were supplemented with 30% bran. It was mainly due to the enthalpy reduction in the starch available for gelatinization when the starch was diluted by the dietary fiber and protein-rich black bean flour. HMT significantly affected the thermal properties of the mix powder. Compared to mix powder, the To, Tp, Tc, and ΔT of HMT-mix powder increased (the gelatinization temperature range, i.e., Tc–To), and the gelatinization enthalpy (ΔH) decreased. The gelatinization temperature for HMT-mix powder was approximately 10 °C higher than that of mix powder. To, Tp and Tc are a measure of the crystalline stability and its variation within the starch granules. ΔH reflects the double-helical and crystalline order and is influenced by the amylopectin chain-length distribution and amylose–lipid complexes [11]. On account of the increased relative crystallinity after HMT, it can be speculated that the decreased enthalpy may be mainly attributed to the destruction of amylose–lipid complexes during HMT.

### 3.5. Scanning Electron Microscopy of Flour and Dough

**Figure 3:**
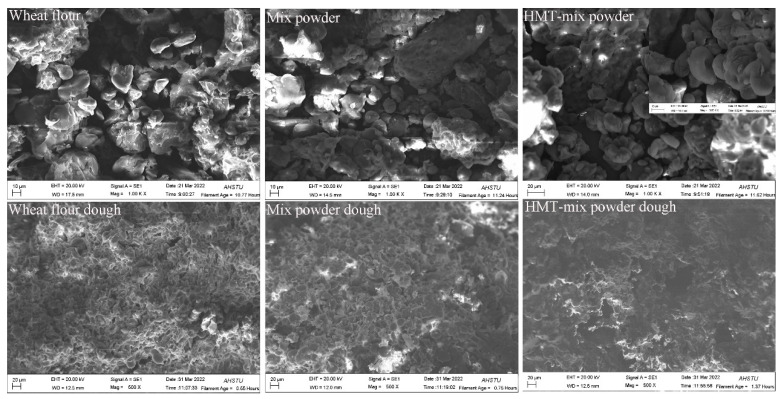
SEM images of wheat four, mix powder, HMT-mix powder and their dough.

Figure 3 shows the SEM images of wheat flour, mix powder, HMT-mix powder, and the corresponding doughs. For the flour or powder samples, the granules varied in the following shapes: round and oval; irregular polyhedral; smooth surface; coarse surface. In addition, granule agglomeration was observed in samples because of their higher water content. HMT was observed to have no apparent effect on the morphology and size of the starch. However, more cracks appeared on the HMT-mix powder granules than on mix powder (as shown in the figure of high multiples), which may be due to the disruption of the weak structures within the granule. The pressure and heating on the starch might cause granules to become a compact form during HMT and even result in cracks on the surface [9]. The same result was observed on breadfruit starch [9].

Compared with the dough of wheat flour, the dough prepared from mix powder displayed a more compact gluten network, in which most wheat starch granules were firmly embedded. In wheat flour dough, some starch granules dissociated with the gluten network. It revealed that adding black soybean powder improved the continuity and stability of gluten, which was also verified by the S–S content of the samples. The higher protein content of black soybean powders contributes to the formation of the gluten network. According to Rakhesh, Fellows, and Sissons [23], the addition of cellulose dilutes starch, and the decreased starch content may affect the starch-protein matrix. The reduction in the amount of starch may facilitate the gluten to encase starch granules. After HMT, the microstructure of the dough showed remarkable changes. Some pores appeared on the HMT-mix powder dough, making its structure loose and reducing its density. The destroyed granules during HMT affected water absorption, further influencing the moisture competition between the starch granules and gluten protein and finally affecting the dough structure.

### 3.6. Content of Free Sulfhydryl Groups and Disulfide Bonds in the Gluten

Free SH and S–S significantly affect the structure and functional characteristics of the dough [24]. S–S bonds play a key role in maintaining the stability of the gluten structure and determining the rheological properties of the dough [25]. The contents of free SH and S–S in the gluten from the dough of wheat flour, mix powder, and HMT-mix powder are shown in Table 4. The contents of free SH and S–S in the gluten for the wheat flour were 3.13 μmol/g and 13.74 μmol/g, respectively, lower than that in the gluten of mix powder. The contents of free SH and S–S in the gluten for the wheat flour were 3.13 μmol/g and 13.74 μmol/g, respectively. The higher S–S content in the mix powder may originate from the significantly higher protein content than the wheat flour (Table 1). These results indicate that the addition of black soybean flour significantly altered the functional and structural characteristics of gluten, especially its stability. Furthermore, HMT affected the content of free sulfhydryl groups and disulfide bonds in the gluten. Compared to mix powder dough, the free SH content in the dough of HMT-mix powder decreased to 4.04 μmol/g, while the S–S content increased to 28.36 μmol/g. Cao et al. [18] reported that the higher content of free SH was mainly caused by the breakage of gluten. As a result, the decrease in free SH content mainly originated from the less breakage of disulfide bonds, indicating that HMT contributes to the formation of disulfide bonds and improves the stability of the dough.

### 3.7. Secondary Structure of the Gluten Network

**Figure 4:**
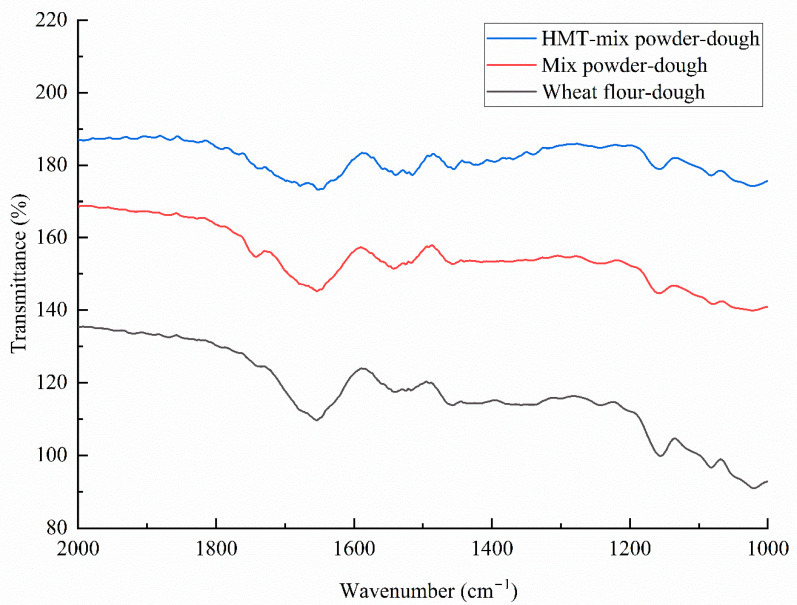
FTIR spectra of dough from wheat flour, mix powders and HMT-mix powder.

The FTIR spectrum of gluten from the mix powders and wheat flour dough is shown in Figure 4. The software PeakFit was used to fit the second derivative of the FTIR spectrum. The secondary structure of gluten was quantified, and the results were summarized in Table 5. Compared with the gluten in the wheat flour dough, the addition of black soybean powders had no significant effect on the α-helix and random coil content. However, the black soybean powder significantly affected the secondary β-sheet and β-turn content. The β-sheet content decreased from 38.45% in wheat flour to 40.66% in mix powder, while the β-turn presented the opposite trend, which increased from 16.37% in wheat flour to 18.36% in mix powder. HMT affected the secondary structure of the gluten network. Compared to mix powder dough, HMT-mix powder dough showed a decrease in α-helix from 19.69 to 18.20 and in β-turn from 18.36 to 16.56. In contrast, the β-sheet increased from 38.45 to 41.90. Additionally, the β-sheet structure is the dominant secondary structure of gluten in all the samples. This result was inconsistent with the conclusion of Bock and Damodaran [26] that β-turn is the preferred secondary structure of gluten in the dough with bran. The differences in sample preparation might lead to different degrees of hydration. In addition, HMT had no effect on the random coil. According to Dong et al. [27], the ordered structure decreased as the random coil content increased. Thus, the ordered structure of the dough was unaffected during HMT.

### 3.8. Amino Acid Composition and Evaluation

The amino acid composition of the cookies is presented in Table 6. For non-essential amino acids (NEAA), incorporating black soybean flour significantly increased the NEAA content compared to wheat flour cookies, except for glutamic acid content. This increase was due to the higher aspartic acid, serine, glycine arginine, and alanine in black soybeans. The glutamic acid had the highest content in wheat flour and mix powder cookies, ranging from 10.91 to 10.98 g·kg^−1^. This result was consistent with the findings of Zhao et al. [20] that the glutamic acid content was the highest in wheat flour. For semi-essential and essential amino acids, most of them also increased with the addition of black soybean flour, indicating that the amino acid composition of black soybean protein is more balanced than that of wheat flour protein. This result was consistent with the findings of Luhovyy et al. [28] that the addition of pulse flour to wheat flour improves the amino acid profile of baked products. Lysine was the limiting amino acid in wheat flour, which limited the availability of other essential and non-essential amino acids. The lysine content of mix powder cookies (5.07 g·kg^−1^) was significantly greater than that of wheat flour cookies (2.42 g·kg^−1^). Thus, the addition of black soybean flour effectively increased the content of limited amino acids in wheat flour cookies and promoted protein complementation. This result is important for improving the amino acid composition of ordinary wheat flour cookies.

Based on Table 6, it was obvious that the HMT dramatically increased the amino acid content of cookies, except for a slight decrease in cysteine and methionine. The glutamic acid increased from 10.98 g·kg^−1^ in mix powder cookies to 34.80 g·kg^−1^ in HMT-mix powder cookies, and leucine increased from 3.20 g·kg^−1^ to 11.13 g·kg^−1^, both more than threefold. The tyrosine increased from 2.15 g·kg^−1^ in mix powder cookies to 6.10 g·kg^−1^ in HMT-mix powder cookies, and proline increased from 5.58 g·kg^−1^ to 14.99 g·kg^−1^, both by more than two times. These results could be explained as follows: phytic acid, condensed tannins, and polyphenols may interact with proteins to form complexes that reduce protein solubility, and thus affect the action of proteases on unstable peptides bonds. Furthermore, the increase in bioavailability of amino acids after HMT may be caused by the reduction or removal of the above antinutritional factors, which increases protein digestibility. Embaby [29] found that all heat treatments significantly reduced the levels of the investigated antinutrients. Another reason was that during HMT, the protein was decomposed by heat, increasing its endogenous amino acid release. The chemical score analysis showed that the chemical score (CS) increased from 9.14% in wheat flour cookies to 12.35% in mix powder cookies, demonstrating that the protein quality of black soybean cookies was significantly greater than that of wheat flour cookies. After HMT, the CS further improved, up to 19.64% in HMT-mix powder cookies. This result demonstrated that the HMT could effectively improve the nutritional value of cookies.

### 3.9. Physical Characteristics Evaluation of Cookies

The weight, diameter, thickness, spread ratio, specific volume, and baking loss of the cookies, as affected by the particle size of black soybean flour, are shown in Table 7. The average weight of cookies varied between 5.77 and 7.56 g, presenting an irregular trend, mainly attributed to the different initial weights of the cookie dough before baking. The addition of black soybean did not affect the diameter of the cookies. However, the thickness decreased from 0.47 cm in wheat flour cookies to 0.35 cm in mix powder cookies, while the spread ratio increased from 10.87 in wheat flour cookies to 14.72 in mix powder cookies. As reported by Korese et al. [30], the content of gluten proteins affected the dough viscosity, and the gluten network helped increase the dough viscosity and decrease the dough spread. Thus, the decrease in thickness and increase in spread ratio were mainly due to the dilution effect of black soybean flour on gluten, resulting in a decrease in dough viscosity, decreased thickness, and increased spread ratio of the cookies. Furthermore, the addition of black soybean flour significantly reduced the baking loss, which may be due to the higher water retention capacity of black soybean flour. After HMT, the physical characteristics of cookies significantly changed.

Compared to the mix powder cookies, the thickness, density, and baking loss of the HMT-mix powder cookies increased, and their spread ratio decreased. The increased baking loss of HMT-mix powder cookies can be explained by the changes in the microstructure of the dough. Based on SEM analysis, the dough of HMT-mix powder presented some pores, and the structure became loose and less dense. These changes may contribute to the water flow and evaporation during baking.

### 3.10. In Vitro Starch Digestibility

Table 8 shows the portions of RDS, SDS, and RS of cookies made from wheat flour, mix powder, and HMT-mix powder. The incorporation of black soybean significantly affected the digestibility of cookies. The mix powder cookies presented lower RDS (32.39%) content, higher SDS (45.97%) content, and higher RS (21.64%) content than wheat flour cookies (34.55%, 44.44%, and 20.46%, respectively). This result was possibly caused by the combination of dietary fiber in black soybean flour with proteins. This combination formed a matrix barrier that surrounded the starch granules, preventing the starch from being hydrolyzed by the enzyme [31]. After HMT, the starch digestion was significantly inhibited. It can be observed that HMT decreased the RDS content but increased the SDS and RS contents. The RDS of starch decreased from 32.39% in mix powder cookies to 23.82% in HMT-mix powder, while the SDS increased from 45.97% to 49.31%, and RS increased from 21.64% to 26.87%. The result was consistent with the findings of Tan et al. [9] that HMT increased the SDS and RS contents of breadfruit starch. The increase in the SDS and RS contents in cookies after HMT may be associated with the changes in starch structure. Xie et al. [11] reported that for starch granules, the crystalline fraction of starch was a primary factor limiting the rate and extent of α-amylase hydrolysis of the starch. They also mentioned that the increase in the RS content by HMT can be attributed to higher crystallinity and increased interactions between amylose and amylopectin. According to the XRD results (Figure 1), HMT increased the relative crystallinity from 20.40% in mix powder to 26.02% in HMT-mix powder. The RDS content was decreased, and SDS and RS contents were increased in HMT-mix powder because of the increase in starch crystallinity during HMT.

### 3.11. Textural Properties Measurements

The textural properties of the cookies made from wheat flour, mix powder, and HMT-mix powder are presented in Table 9. Compared to wheat flour cookies, the addition of black soybean flour dramatically increased the hardness of the cookies up to 13.86 N. This increase was related to the higher protein content in mix powder cookies, as Cappa et al. [8] reported that increased protein content could make the cookies harder. The hardness of HMT-mix powder cookies was 7.05 N, which was significantly lower than mix powder cookies. The dough of HMT-mix powder presented a porous and loose structure, decreasing the hardness of the cookies. The fracturability of mix powder cookies decreased compared to wheat flour cookies, which increased up to 1.32 g after HMT. According to Korese et al. [30], the fracturability of the cookies negatively correlates with their crispness. Therefore, HMT can cause a decrease in the crispness of cookies, which helps avoid potential difficulties in industrial handling (e.g., packaging and transportation phases). Compared to wheat flour cookies, the incorporation of black soybean flour significantly increased the gumminess value, while HMT decreased the gumminess value. Generally, the proper reduction in gumminess was beneficial to the quality of the cookies. The chewiness of the cookies decreased with the incorporation of black soybean flour. It increased from 6.30 mJ in wheat flour cookies to 11.70 mJ in mix powder cookies. In addition, HMT had no significant effect on chewiness. Low chewiness can lead to a delicate taste and sticky texture of cookies. Overall, the texture analysis demonstrated that HMT improved the texture of cookies.

## 4. Conclusions

In this study, HMT was used to treat the mix-powder of wheat flour and black soybean to improve the nutritional value and reduce the starch digestibility of cookies. The physicochemical properties, structural characteristics of the flour samples and corresponding dough, and physical, nutritional, and textural properties of the cookies were investigated. The results showed that HMT could significantly vary the properties and structure of the dough and cookies. After HMT, the water retention, lactic acid retention capacity, and oil binding capacity of the mix powder dramatically increased. The gelatinization temperature (To, Tp and Tc) increased by approximately 10 °C, while ΔH decreased. The morphology and size of the granules were not affected by HMT, but some cracks and pores appeared on the HMT-mix powder granules and HMT-mix powder dough, respectively. HMT facilitated the formation of disulfide bonds and improved the stability of the dough. The amino acid analysis showed that HMT significantly increased the chemical score of the cookies. It increased from 12.35% in mix powder-cookies to 19.64% in HMT-mix powder cookies. Notably, HMT dramatically decreased the in vitro digestion characteristics of starch. These results suggest that HMT can be used as an effective ‘green’ process for increasing the total SDS content, RS content, and chemical score of cookies with superfine black soybean flour. In addition, HMT can effectively improve the nutritional value of cookies without negative effects on the dough’s processing properties and the cookies’ physical properties. Therefore, this study can provide guidance for the applications of HMT-superfine black soybean flour in baked foods. Considering that starch is the main component of all ingredients (wheat flour and mix powders) in this paper, their starch digestibility was emphasized; while protein was another important component, its role in the whole digestion process was unfortunately ignored, which is worthy of further study as one of the research directions.

## Figures and Tables

**Table 1:** Proximate composition of black soybean powder and wheat flour.

Sample	Moisture (g·kg^−1^)	Ash (g·kg^−1^)	Protein (g·kg^−1^)	Lipid (g·kg^−1^)	Dietary Fiber (g·kg^−1^)
Wheat flour	1.28 ± 0.02 ^b^	0.06 ± 0.01 ^c^	1.67 ± 0.01 ^c^	0.90 ± 0.05 ^c^	0.13 ± 0.02 ^c^
Mix powder	1.42 ± 0.01 ^a^	0.21 ± 0.01 ^a^	2.64 ± 0.01 ^a^	1.65 ± 0.04 ^a^	0.54 ± 0.02 ^a^
HMT-mix powder	1.13 ± 0.01 ^c^	0.19 ± 0.00 ^b^	2.55 ± 0.00 ^b^	1.52 ± 0.04 ^b^	0.51 ± 0.01 ^b^

^a–c^ Data are means of triplicate analyses with standard deviation. Means in the same column with different lowercase letters were significantly different (*p* < 0.05).

**Table 2:** Water retention (WRC), lactic acid retention (LARC) and oil binding capacities (OBC) of wheat flour, mix powder and HMT-mix powder.

Sample	WRC (g/g)	LARC (g/g)	OBC (g/g)
Wheat flour	0.64 ± 0.01 ^c^	0.95 ± 0.03 ^b^	1.00 ± 0.04 ^b^
Mix powder	0.76 ± 0.01 ^b^	0.66 ± 0.01 ^c^	0.95 ± 0.04 ^c^
HMT-mix powder	1.38 ± 0.05 ^a^	1.70 ± 0.01 ^a^	1.50 ± 0.04 ^a^

^a–c^ Data are means of triplicate analyses with standard deviation. Means in the same column with different lowercase letters were significantly different (*p* < 0.05).

**Table 3:** Thermal properties of wheat flour, mix powder and HMT-mix powder.

Sample	To (°C)	Tp (°C)	Tc (°C)	ΔT (°C)	ΔHg (J/g)
Wheat flour	57.75 ± 0.03 ^c^	62.45 ± 0.00 ^c^	66.98 ± 0.02 ^c^	9.23 ± 0.02 ^b^	5.28 ± 0.03 ^a^
Mix powder	59.74 ± 0.01 ^b^	63.97 ± 0.00 ^b^	68.66 ± 0.01 ^b^	8.92 ± 0.00 ^c^	3.17 ± 0.01 ^b^
HMT-mix powder	69.06 ± 0.07 ^a^	74.12 ± 0.00 ^a^	78.82 ± 0.05 ^a^	9.76 ± 0.04 ^a^	2.03 ± 0.03 ^c^

^a–c^ Data are means of triplicate analyses with standard deviation. Means in the same column with different lowercase letters were significantly different (*p* < 0.05).

**Table 4:** Total sulfhydryl groups (total SH), free sulfhydryl groups (free SH) and disulfide bonds (S–S) of dough from wheat flour, mix powder and HMT-mix powder.

Samples	Total SH (μmol/g)	Free SH (μmol/g)	S–S (μmol/g)
Wheat flour dough	30.62 ± 0.27 ^c^	3.13 ± 0.05 ^c^	13.74 ± 0.16 ^c^
Mix powder dough	49.25 ± 0.16 ^b^	5.70 ± 0.10 ^a^	21.77 ± 0.12 ^b^
HMT-mix powder dough	60.75 ± 0.20 ^a^	4.04 ± 0.02 ^b^	28.36 ± 0.11 ^a^

^a–c^ Data are means of triplicate analyses with standard deviation. Means in the same column with different lowercase letters were significantly different (*p* < 0.05).

**Table 5:** Secondary structure content of gluten from dough of wheat flour, mix powder and HMT-mix powder.

Dough Samples	α-Helix	β-Sheet	β-Turn	Random Coil
Wheat flour	19.94 ± 0.35 ^a^	40.66 ± 0.45 ^a^	16.37 ± 0.86 ^b^	23.02 ± 0.96 ^a^
Mix powder	19.69 ± 0.52 ^a^	38.45 ± 0.50 ^b^	18.36 ± 0.68 ^a^	23.49 ± 0.44 ^a^
HMT-mix powder	18.20 ± 0.10 ^b^	41.90 ± 1.00 ^a^	16.56 ± 1.17 ^b^	23.35 ± 0.36 ^a^

^a,b^ Data are means of triplicate analyses with standard deviation. Means in the same column with different lowercase letters were significantly different (*p* < 0.05).

**Table 6:** Amino acid composition of cookies as influenced by particle size of black soybean flour (g·kg^−1^ cookies).

Sample	Wheat Flour-Cookies	Mix Powder-Cookies	HMT-Mix Powder-Cookies
Non-essential amino acids			
Aspartic acid	4.17 ± 0.01 ^c^	9.14 ± 0.03 ^b^	13.68 ± 0.04 ^a^
Glutamic acid	10.98 ± 0.01 ^b^	10.91 ± 0.01 ^b^	34.80 ± 0.02 ^a^
Serine	3.93 ± 0.03 ^c^	5.20 ± 0.03 ^b^	7.75 ± 0.05 ^a^
Glycine	2.88 ± 0.02 ^c^	4.46 ± 0.01 ^b^	6.16 ± 0.02 ^a^
Arginine	3.06 ± 0.02 ^c^	6.42 ± 0.01 ^b^	8.61 ± 0.03 ^a^
Alanine	3.01 ± 0.01 ^c^	4.85 ± 0.01 ^b^	10.26 ± 0.02 ^a^
Semi-essential and essential amino acids			
Threonine	2.59 ± 0.01 ^c^	4.31 ± 0.01 ^b^	6.57 ± 0.03 ^a^
Cystine	0.78 ± 0.01 ^c^	1.10 ± 0.02 ^a^	1.02 ± 0.02 ^b^
Valine	3.70 ± 0.01 ^c^	5.04 ± 0.01 ^b^	6.67 ± 0.01 ^a^
Methionine	1.14 ±0.01 ^c^	1.40 ± 0.01 ^a^	1.31 ± 0.04 ^b^
Isoleucine	3.18 ± 0.01 ^c^	5.04 ± 0.01 ^b^	6.06 ± 0.05 ^a^
Leucine	6.07 ± 0.02 ^b^	3.20 ± 0.02 ^c^	11.13±0.05 ^a^
Tyrosine	1.13 ± 0.01 ^c^	2.15 ± 0.01 ^b^	6.10 ± 0.03 ^a^
Phenylalanine	4.13± 0.01 ^c^	6.22 ± 0.01 ^b^	7.38 ± 0.03 ^a^
Lysine	2.42 ± 0.01 ^c^	5.07 ± 0.01 ^b^	5.70 ± 0.01 ^a^
Histidine	1.87± 0.01 ^c^	3.02 ± 0.01 ^b^	5.43 ± 0.05 ^a^
Proline	5.44 ± 0.01^c^	5.58 ± 0.01 ^b^	14.99 ± 0.07 ^a^
CS (%)	9.14 ± 0.01^c^	12.35 ± 0.02 ^b^	19.64 ± 0.06 ^a^

^a–c^ Data are means of triplicate analyses with standard deviation. Means in the same line with different lowercase letters were significantly different (*p* < 0.05).

**Table 7:** Physical properties of cookies made from wheat four, mix powder and HMT-mix powder.

Sample	Weight (10^−3^ kg)	Diameter (cm)	Thickness (cm)	Spread Ratio (%)	Density (g/cm^3^)	Baking Loss (%)
Wheat flour-cookies	6.07 ± 0.12 ^b^	5.11 ± 0.01 ^a^	0.47 ± 0.00 ^a^	10.87 ± 0.02 ^c^	0.63 ± 0.02 ^c^	14.69 ± 0.07 ^a^
Mix powder-cookies	5.77 ± 0.30 ^b^	5.16 ± 0.05 ^a^	0.35 ± 0.00 ^c^	14.72 ± 0.08 ^a^	0.79 ± 0.07 ^b^	10.67 ± 0.27 ^c^
HMT-mix powder-cookies	7.56 ± 0.06 ^a^	4.98 ± 0.03 ^b^	0.42 ± 0.02 ^b^	11.97 ± 0.42 ^b^	0.92 ± 0.03 ^a^	13.14 ± 0.26 ^b^

^a–c^ Data are means of triplicate analyses with standard deviation. Means in the same column with different lowercase letters were significantly different (*p* < 0.05).

**Table 8:** Englyst digestion values of starch fractions of cookies made from wheat flour, mix powder and HMT-mix powder.

Sample	RDS (%)	SDS (%)	RS (%)
Wheat flour-cookies	34.55 ± 0.53 ^a^	44.99 ± 0.12 ^c^	20.46 ± 0.53 ^b^
Mix powder-cookies	32.39 ± 0.55 ^b^	45.97 ± 0.42 ^b^	21.64 ± 0.91 ^b^
HMT-mix powder-cookies	23.82 ± 0.42 ^c^	49.31 ± 0.55 ^a^	26.87 ± 0.75 ^a^

^a–c^ Data are means of triplicate analyses with standard deviation. Means in the same column with different uppercase letters were significantly different (*p* < 0.05).

**Table 9:** Textural properties of wheat flour cookies, mix powder cookies and HMT-mix powder cookies.

Sample	Hardness (N)	Fracturability (g)	Gumminess (g)	Chewiness (mJ)
Wheat flour Cookie	6.81 ± 0.01 ^b^	1.16 ± 0.04 ^b^	559.67 ± 5.77 ^c^	6.30 ± 0.10 ^b^
Mix powder Cookie	13.86 ± 0.07 ^a^	1.02 ± 0.01 ^c^	984.33 ± 8.50 ^a^	11.70 ± 0.61 ^a^
HMT-mix powder Cookie	7.05 ± 0.68 ^b^	1.32 ± 0.06 ^a^	828.00 ± 21.79 ^b^	12.17 ± 1.00 ^a^

^a–c^ Data are means of triplicate analyses with standard deviation. Means in the same column with different lowercase letters were significantly different (*p* < 0.05).

## Data Availability

No new data were created or analyzed in this study. Data sharing is not applicable to this article.

## References

[1] Culetu A., Stoica-Guzun A., Duta D.E. (2021). Impact of fat types on the rheological and textural properties of gluten-free oat dough and cookie. Int. J. Food Sci. Technol..

[2] Hooda S., Jood S. (2005). Organoleptic and nutritional evaluation of wheat biscuits supplemented with untreated and treated fenugreek flour. Food Chem..

[3] Wang L., Li S.L., Gao Q.Y. (2014). Effect of resistant starch as dietary fiber substitute on cookies quality evaluation. Food Sci. Technol. Res..

[4] Sozer N., Cicerelli L., Heiniö R.L., Poutanen K. (2014). Effect of wheat bran addition on in vitro starch digestibility, physicomechanical and sensory properties of biscuits. J. Cereal Sci..

[5] Vasanthakumari P., Jaganmohan R. (2018). Process development and formulation of multi-cereal and legume cookies. J. Food Process. Preserv..

[6] Luo D., Liang X., Xu B., Kou X., Li P., Han S., Liu J., Zhou L. (2017). Effect of inulin with different degree of polymerization on plain wheat dough rheology and the quality of steamed bread. J. Cereal Sci..

[7] Zhao B., Deng J., Li M., Li H., Zhang Y., Gong H., Chen Z. (2020). Preparation and quality evaluation of potato steamed bread with wheat gluten. Food Sci. Nutr..

[8] Cappa C., Kelly J.D., Ng P.K.W. (2020). Baking performance of 25 edible dry bean powders: Correlation between cookie quality and rapid test indices. Food Chem..

[9] Tan X., Li X., Chen L., Xie F., Li L., Huang J. (2017). Effect of heat-moisture treatment on multi-scale structures and physicochemical properties of breadfruit starch. Carbohydr. Polym..

[10] Rizkalla S.W., Bellisle F., Slama G. (2002). Health benefits of low glycaemic index foods, such as pulse in diabetic patients and healthy individuals. Br. J. Nutr..

[11] Xie H.F., Gao J.Y., Xiong X.Y., Gao Q.Y. (2018). Effect of heat-moisture treatment on the physicochemical properties and in vitro digestibility of the starch-guar complex of maize starch with varying amylose content. Food Hydrocoll..

[12] Na J.H., Kim H.R., Kim Y., Lee J.S., Park H.J., Moon T.W., Lee C.J. (2020). Structural characteristics of low-digestible sweet potato starch prepared by heat-moisture treatment. Int. J. Biol. Macromol..

[13] Wang H., Liu Y., Chen L., Li X., Wang J., Xie F. (2018). Insights into the multi-scale structure and digestibility of heat-moisture treated rice starch. Food Chem..

[14] AACC (2000). Approved Methods of the AACC.

[15] Yang L.P., Liu Y., Yang J.T., Du C.L., Zhai L.G. (2021). Changes in the multi-scale structure and physicochemical properties of starch during potato growth. J. Sci. Food Agric..

[16] Xu X.J., Luo Z.G., Yang Q.Y., Xiao Z.G., Lu X.X. (2019). Effect of quinoa flour on baking performance, antioxidant properties and digestibility of wheat bread. Food Chem..

[17] Bressiani J., Oro T., Santetti G.S., Almeida J.L., Bertolin T.E., Gómez M., Gutkoski L.C. (2017). Properties of whole grain wheat flour and performance in bakery products as a function of particle size. J. Cereal Sci..

[18] Cao Y.F., Zhang M., Dong S., Guo P., Li H.J. (2020). Impact of potato pulp on the processing characteristics and gluten structures of wheat flour dough. J. Food Process. Preserv..

[19] Sulieman A.A., Zhu K.X., Peng W., Hassan H.A., Obadi M., Siddeeg A., Zhou H.M. (2019). Rheological and quality characteristics of composite gluten-free dough and biscuits supplemented with fermented and unfermented Agaricus bisporus polysaccharide flour. Food Chem..

[20] Zhao Z., Mu T., Sun H.N. (2019). Comparative study of the nutritional quality of potato steamed bread fermented by different sourdoughs. J. Food Process. Preserv..

[21] Hera E.D.L., Rosell C.M., Gomez M. (2014). Effect of water content and flour particle size on gluten-free bread quality and digestibility. Food Chem..

[22] Lapčíková B., Burešová I., Lapčík L., Dabash V., Valenta T. (2019). Impact of particle size on wheat dough and bread characteristics. Food Chem..

[23] Rakhesh N., Fellows C.M., Sissons M. (2015). Evaluation of the technological and sensory properties of durum wheat spaghetti enriched with different dietary fibres. J. Sci. Food Agric..

[24] Chen G., Hu R., Li Y. (2018). Potassium chloride affects gluten microstructures and dough characteristics similarly as sodium chloride. J. Cereal Sci..

[25] Delcour J.A., Joye I.J., Pareyt B., Wilderjans E., Brijs K., Lagrain B. (2012). Wheat gluten functionality as a quality determinant in cereal-based food products. LWT Food Sci. Technol..

[26] Huang Y.C., Lai H.M. (2010). Noodle quality affected by different cereal starches. J. Food Eng..

[27] Dong Y., Lan T., Wang X., Zhang Y., Jiang L., Sui X. (2020). Preparation and characterization of soy protein microspheres using amorphous calcium carbonate cores. Food Hydrocoll..

[28] Luhovyy B.L., Hamilton A., Kathirvel P., Mustafaalsaafin H. (2017). The effect of navy bean flour particle size on carbohydrate digestion rate measured in vitro. Cereal Foods World.

[29] Embaby H.E.S. (2011). Effect of heat treatments on certain antinutrients and in vitro protein digestibility of peanut and sesame seeds. Food Sci. Technol. Res..

[30] Korese J.K., Chikpah S.K., Hensel O., Pawelzik E., Sturm B. (2021). Efect of orange-feshed sweet potato four particle size and degree of wheat four substitution on physical, nutritional, textural and sensory properties of cookies. Eur. Food Res. Technol..

[31] Foschia M., Peressini D., Sensidoni A., Brennan M.A., Brennan C.S. (2015). Synergistic effect of different dietary fibres in pasta on in vitro starch digestion?. Food Chem..

